# The Effects of the Healthy Primary School of the Future on Children’s Fruit and Vegetable Preferences, Familiarity and Intake

**DOI:** 10.3390/nu13093241

**Published:** 2021-09-17

**Authors:** Marla T. H. Hahnraths, Maartje Willeboordse, Patricia van Assema, Bjorn Winkens, Constant P. van Schayck

**Affiliations:** 1 Department of Family Medicine, Care and Public Health Research Institute (CAPHRI), Maastricht University, P.O. Box 616, 6200 MD Maastricht, The Netherlands; maartje.willeboordse@maastrichtuniversity.nl (M.W.); onno.vanschayck@maastrichtuniversity.nl (C.P.v.S.); 2 Department of Health Promotion, Care and Public Health Research Institute (CAPHRI), School of Nutrition and Translational Research in Metabolism (NUTRIM), Maastricht University, P.O. Box 616, 6200 MD Maastricht, The Netherlands; p.vanassema@maastrichtuniversity.nl; 3 Department of Methodology and Statistics, Care and Public Health Research Institute (CAPHRI), Maastricht University, P.O. Box 616, 6200 MD Maastricht, The Netherlands; bjorn.winkens@maastrichtuniversity.nl

**Keywords:** primary school, health promoting school, nutrition, eating habits, repeated exposure, taste preferences, familiarity

## Abstract

Mere exposure is an often-described strategy to increase children’s food familiarity, preferences, and intake. Research investigating this method in less controlled settings is scarce. This study investigates the effects of repeated fruit and vegetable (FV) exposure through the Healthy Primary School of the Future (HPSF) on children’s FV familiarity, preferences, and intake. The study had a longitudinal quasi-experimental design comparing two full HPSFs (focus: nutrition and physical activity) with two partial HPSFs (focus: physical activity) in the Netherlands. Annual measurements (child-reported questionnaires) were conducted during 2015–2019 in 833 7–12-year-old children. The study was registered on ClinicalTrials.gov (NCT02800616). After correction for baseline, full HPSFs had, on average, a lower number of unfamiliar vegetable items after one (effect size (ES) = −0.28) and three years (ES = −0.35) and a higher number of disliked vegetable items after one year (ES = 0.24) than partial HPSFs. Unfavorable intervention effects were observed for fruit intake after one (odds ratio (OR) = 0.609) and four years (OR = 0.451). Repeated FV exposure had limited effects on children’s FV familiarity, preferences, and intake, likely due to insufficient taste exposure. Considering the widespread implementation of school-based mere exposure efforts, it is highly relevant to further investigate under which circumstances mere exposure effectively contributes to improvements in (determinants of) FV intake.

## 1. Introduction

Despite the important health benefits of fruit and vegetable (FV) intake, insufficient FV consumption is a global issue related to multiple health problems, such as obesity, coronary heart disease, stroke, and cancer [1,2]. In 2017, globally, 3.9 million deaths could be attributed to inadequate FV consumption [2]. As lifestyle behaviors that are formed during childhood are likely to persist throughout adulthood, promoting FV consumption at a young age is expected to result in both immediate as well as long-term health benefits [3,4,5]. In the Netherlands, the current dietary habits of children show significant room for improvement as their FV intake is currently suboptimal. Over the period of 2014–2016, only 42% of children (aged 4–9 years) consumed at least recommended 150 g of fruit per day in the Netherlands; this percentage dropped to 20% for 9–12-year-olds. The percentages for vegetable intake were comparable: 41% of 4–9-year-olds and 25% of 9–12-year-olds consumed at least 150 g of vegetables per day [6].

Food preferences or the related concept of liking have been identified as strong predictors of food intake in children, and this relationship has also been described for FV specifically [7,8,9,10,11,12]. Food preferences are defined as learned dispositions that are the result of children’s experience with a food [10,13]. One of the strongest determinants of children’s food preferences is therefore their familiarity with a specific food [10,14,15]. An often-described strategy to increase liking of a food is mere exposure; various studies have shown that repeated exposure to and consumption of a food (i.a., FV) enhances familiarity and subsequently increase liking [14,16,17,18,19]. Exposure to the food in a positive social context where others are also consuming it is thought to amplify this process [20,21].

The number of exposures required to impact preferences for a food seems to increase with age. While the preferences of 2- to 4-year-olds were often increased after five to fifteen exposures [22,23,24], more exposures seem necessary to influence 5- to 12-year-olds’ preferences, although the exact number of exposures varies across studies [25,26]. Mere exposure therefore seems to be a particularly powerful strategy to form and impact food preferences in very young children (0–4 years), but research focusing on its effects in older children (4–12 years) is relatively scarce [19].

The school environment is a promising setting to influence the dietary habits of children, as, at school, children from various backgrounds come together on a regular basis for several critical developmental years [27,28]. Various school-based interventions aiming to increase FV preferences through repeated exposure (e.g., by FV delivery schemes or tasting sessions) have been implemented and showed promising results. In studies by Lakkakula et al. and Schindler et al., repeated FV exposure through school-based interventions resulted in increases in vegetable liking and willingness to try fruit [29,30]. However, as these studies often tightly control children’s exposure to FV or are of short duration, there is a need for large, long-term studies in less controlled settings [29,30]. 

The Dutch initiative ‘Healthy Primary School of the Future’ (HPSF) is a multicomponent programme that was implemented at several primary schools. The aim of HPSF was to sustainably integrate health promotion within the school system, thereby improving children’s health and well-being over the long term. For a period of four years, two ‘full HPSFs’ implemented (1) various interventions aimed at increasing children’s exposure to FV (provision of a daily healthy school lunch and mid-morning snack), and (2) structured physical activity (PA) sessions during lunch break time. Two ‘partial HPSFs’ implemented solely the structured PA sessions [31]. The current study is part of the overall HPSF research project executed by a large multidisciplinary research team. Previous studies reported on the study design [31], research approach [32], process evaluation [33], non-response, and external validity [34]. Furthermore, various reports of this project revealed positive intervention effects on various health-related outcomes in children (e.g., BMI z-score, waist circumference, PA, and dietary behaviors) [35,36,37,38].

**Figure 1:**
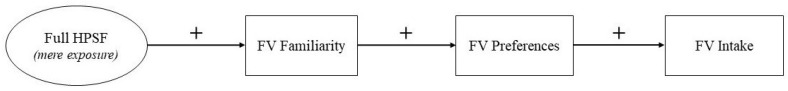
Hypothesized working mechanism of the full HPSF intervention. + Indicates a positive relationship between the concepts.

The present study aims to answer the following research question: What are the effects of one- to four-year exposure to school-based FV exposure interventions on children’s familiarity with and preferences for specific FV items, and on children’s FV intake? It was hypothesized that children’s regular, increased exposure to FV in the full HPSFs would lead to an increase in the number of familiar and liked FV items and subsequently to an increase in FV intake (Figure 1). Furthermore, it was hypothesized that longer exposure to the full HPSF intervention, and therefore a higher number of FV exposures, would lead to more prominent effects.

## 2. Materials and Methods

### 2.1. Study Design

The overall project had a longitudinal quasi-experimental design with two full intervention schools (full HPSFs), two partial intervention schools (partial HPSFs), and four control schools (not discussed in the current paper), which were all recruited based on voluntary participation [31]. All participating schools were members of the educational board MOVARE and located in the Parkstad region. Parkstad is a region located in the southern part of the Netherlands that consists of eight municipalities (Heerlen, Kerkrade, Landgraaf, Brunssum, Simpelveld, Voerendaal, Nuth, and Onderbanken). Compared to other areas in the Netherlands, Parkstad can be defined as a low to moderate socioeconomic area, characterized by a high prevalence of chronic diseases and a low life expectancy [34,39]. 

All HPSFs started implementation of the full or partial HPSF intervention in November 2015. Annual measurements were conducted in various ways by trained researchers in September–November of 2015 (T0), 2016 (T1), 2017 (T2), 2018 (T3), and 2019 (T4) during one week of measurements per school [31]. The study had a dynamic open cohort, meaning that enrolled students could participate at any moment during the study duration (between 2015 and 2019). This could be children who were already enrolled in one of the schools or children who had newly entered school at some point during the study duration (either at four years old or at a later age). Recruitment was done via information brochures and classroom visits. All participants were required to complete an informed consent form, signed by both parents/caregivers, and by children if they were aged twelve years or older. Participants were excluded when they left school. If they switched to other participating schools during the study period, only data from the original school were included in the analyses. The study was registered in the ClinicalTrials.gov database in June 2016 (NCT02800616).

### 2.2. The Healthy Primary School of the Future

HPSF was developed by the regional educational board MOVARE, the regional public health services, and Maastricht University [31]. The intervention consisted of two main changes: (1) daily provision of a free healthy school lunch and mid-morning snack, and (2) daily structured PA sessions after lunch. Two schools implemented the complete HPSF intervention (full HPSFs), while two other schools implemented solely the structured PA sessions (partial HPSFs). Four control schools that continued with their regular curriculum that is common practice in the Netherlands were also included in the overall study. However, these schools were not discussed in the present study and a comparison was made between the full HPSFs and the partial HPSFs only. This made it possible to investigate the effects of the provided healthy school lunch and mid-morning snack without potentially contaminating the results with effects caused by the PA sessions.

In all intervention schools, lunch break time was prolonged with 45 to 75 min, thereby extending the school day. In full HPSFs, various nutrition-related changes were implemented. The most prominent change was the provision of a daily healthy school lunch. Normally, Dutch primary school pupils bring their lunch from home or go home to eat lunch. For the intervention, a bread-based lunch menu cycle was developed by a dietician and the lunch was provided by catering services. The menu cycle changed every ten weeks and at least 80% of the provided products met the dietary guidelines of the Dutch Health Council [40]. A wide variety of FV items were included in the lunch menu cycles (Table S1). The lunch was offered in a buffet style in the classroom or at a central location in school (depending on the available space within a school), and children were free to compose their own lunch out of the various available food products. Pedagogical staff and teachers encouraged children to include healthy products such as FV in their lunch. For this purpose, they used role modeling (e.g., tasting and consuming healthy products in the presence of children), nudging (e.g., visible placement of ready-to-eat healthy products), rewards for consumption of healthy foods (e.g., coins that could later be exchanged for fun activities), and giving positive attention to children consuming and enjoying healthy foods. These actions were not organized systematically but were largely bottom-up initiatives that occurred in both full HPSFs throughout the project. Additionally, children received a daily healthy mid-morning snack, which, most of the time, was a piece of fruit. After the first intervention year, additional weekly short ‘educational lunches’ were organized, where children learned about a specific food item that was provided during lunch that week (using educational materials inspired by the Taste Lessons program [41] and adapted by the regional public health services to fit the FV items provided during lunch).

Children in both the full and partial HPSFs participated in structured PA and/or cultural sessions during lunch break time. Besides these top-down initiated changes, schools were encouraged to implement additional health-promoting initiatives. Especially towards the end of the project, this resulted in extra changes in the nutritional policies of all intervention schools (e.g., rules regarding healthy birthday treats, snacks, and drinks) [33].

### 2.3. Study Population

All children who were enrolled in full and partial HPSFs between 2015 and 2019 were invited to participate in the overall research project. Due to the study’s dynamic character, recruitment of participants continued throughout the duration of the research project. To answer the current study’s research questions, several inclusion criteria were applied. First, to be able to adequately assess the effects of HPSF’s mere exposure component, only participants who were exposed to the school environment from T0 (baseline) onwards were included in the present study. This included children who enrolled in the study at T0 and children who started study participation at a later moment but were already enrolled in one of the HPSFs at baseline. In this way, exposure to FV items as part of HPSF was equal for all subjects. 

Second, only children from study years four to eight (age range 7–12 years; internationally comparable to grades two to six) were included in the present study, due to age-related differences in questionnaire formulation for younger children (study years one to three). Third, only data of children who had a valid response for at least one outcome during at least one measurement from T1 to T4 were included. 

The present study’s inclusion criteria meant that baseline data were not available for all participants, as some subjects were only included at a later moment in the study (because they were not yet enrolled in the overall study or not yet in study years four to eight at previous timepoint(s)). An overview of the participants included at each timepoint can be found in Table 1.

### 2.4. Data Collection Procedures and Measures

#### 2.4.1. FV Familiarity and Preferences

Participants filled out a paper-based questionnaire during class hours under the guidance of at least one member of the research team. Filling out this questionnaire took approximately 30 min as other aspects such as PA, dietary behavior, and general health were also assessed. FV familiarity and preferences were assessed by twelve fruit items and sixteen vegetable items (Table 2), formulated in the following way: ‘Indicate how much you like the following type of fruit or vegetable’. Each item consisted of the FV item’s name accompanied by its picture. Between T0 and T1, the picture for pear was changed, as problems with the visibility of this picture were observed during questionnaire administration at T0. On a semantic differential rating scale, participants assessed each item as (1) ‘disliked’ (accompanied by a picture of a sad face), (2) ‘neutral’ (accompanied by a picture of a neutral face), or (3) ‘liked’ (accompanied by a picture of a smiley face). If children were unfamiliar with or had never tried an FV item, they could indicate this by checking a fourth response option accompanied by a question mark. This method of assessing FV familiarity and preferences resembled the visual cart-sorting technique that was previously tested and used to assess food and activity patterns in African American girls [42].

Separate familiarity scores (unfamiliar/familiar) and preference scores (disliked/neutral/liked) were composed for the twenty-eight FV items. If a participant had checked one of the three preference response options (‘disliked’, ‘neutral’, ‘liked’), familiarity with the FV item was assumed. If the fourth response option (‘don’t know this FV item/never tried’) was checked, this indicated unfamiliarity with the FV item. When a participant indicated that they were unfamiliar with an FV item, preference for this item was set to missing. To facilitate data analysis, familiarity and preference summary scores were computed for fruit and vegetables. Familiarity summary scores were formed through computing the total number of unfamiliar fruit items and the total number of unfamiliar vegetable items. For preference summary scores, the total number of disliked items was calculated for fruit and vegetables. Summary scores could range from 0 to 12 for fruit and 0 to 16 for vegetables, and were calculated for children with ≤3 missing responses (fruit) and ≤4 missing responses (vegetables) (corresponding to a maximum missing rate of 25%). In case of missing responses, the average of the observed scores was multiplied by the number of items of the scale (twelve for fruit and sixteen for vegetables). This is equivalent to mean imputation, i.e., imputing the mean score of the items from the same scale (fruit or vegetables) and same child.

#### 2.4.2. FV Intake

The child-reported questionnaire also contained questions regarding FV intake, which were used in the present study. The questions were formulated as: ‘Do you consume fruit?’ and ‘Do you consume vegetables?’ (response options ‘(almost) never’, ‘sometimes (1–3 days a week)’, ‘often (4–6 days a week)’, and ‘every day’). This method of assessing FV intake is comparable to methods previously used in similar studies, although questionnaire formulation was slightly changed [42,43].

#### 2.4.3. Socio-Demographic Characteristics

Children’s age and sex were collected via the school database and checked for correctness with data from the regional Youth Health Department. A digital questionnaire was sent out to parents of all participating children to obtain information about the children’s socioeconomic status (SES) and ethnicity. Filling out the questionnaire took approximately 30 min. Other aspects such as PA, dietary behavior, and general health were also assessed. SES was calculated based on standardized scores for maternal and paternal education level and household income (adjusted for household size) [44]. The mean scores were categorized into low, middle, and high SES scores based on tertiles. Children’s ethnicity was determined by the country of birth of both parents and divided into (1) Western background (including the Netherlands, all European countries (except Turkey), Japan, Indonesia, and Oceania), and (2) non-Western background (when at least one parent was born in a non-Western country) [45]. 

### 2.5. Data Processing and Statistical Analyses

Data were analyzed using IBM SPSS Statistics for Windows (version 25.0, IBM Corp, Armonk, NY, USA). Participants were included in the analyses if they were exposed to the school environment at T0 and had a valid response for at least one outcome during at least one measurement from T1 to T4. 

Pearson’s chi-square tests and independent-samples t-tests were conducted to analyze the comparability of observed participant characteristics at T0, i.e., sex, age, SES, ethnicity, FV familiarity, FV preferences, and FV intake, among the full and partial HPSFs. Linear mixed model (LMM) analyses were used to analyze the intervention effects on participants’ FV familiarity and preferences (using the computed summary scores). Ordinal logistic model analyses with exchangeable covariance structure were used for FV intake. For LMM, the best-fitting covariance structure was determined for each analysis, based on the lowest Bayesian Information Criterion (BIC) value. As measurements were repeated within participants, a two-level model with repeated measurements as first level and participants as second level was used. The fixed part of the model consisted of condition (full HPSF, partial HPSF), time (T0–T4), and the interaction term condition × time. All analyses were adjusted for the following covariates: sex (boy/girl), study year (at T0; one to eight), SES (low/middle/high), and ethnicity (Western/non-Western).

For all analyses, a two-sided *p*-value ≤ 0.05 was considered statistically significant. Standardized effect sizes (ES) were determined for continuous outcome variables (FV familiarity and preferences) and were computed as the estimated mean difference divided by the square root of the residual variance at baseline. According to the benchmarks suggested by Cohen et al., ES ≤ 0.2 were considered small, values between 0.2 and 0.5 were considered medium, and ES ≥ 0.5 were considered large [46]. Categorical outcomes (FV intake) resulted in odds ratios (OR).

## 3. Results

### 3.1. Demographic Characteristics

Of the 1243 students exposed to the school environment at T0, 998 (80.3%) handed in a completed informed consent form in 2015–2019. Of these students, 833 (83.5%) were included in the analyses as they met the additional inclusion criteria applied in the present study. Of the included subjects, 397 children (47.7%) started study participation at a later moment but were already enrolled in one of the HPSFs at baseline. A detailed overview of the subjects included at each timepoint can be found in Figure S1.

Table 3 provides an overview of the sample’s baseline characteristics. There were no significant differences in baseline characteristics between full and partial HPSFs. At baseline, participants were familiar with and liked the majority of FV items. Fruit items were more often familiar and liked than vegetable items. A detailed overview of observed mean familiarity and preference scores at baseline for the different FV items can be found in Table S2. Approximately 40% of participants in both the full and partial HPSFs were indicated to consume FV on a daily basis. 

### 3.2. Intervention Effects on FV Familiarity and Preferences

**Figure 2:**
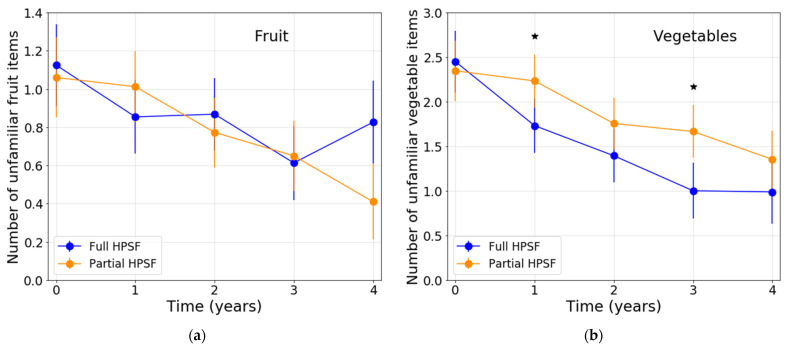
Estimated means of children’s fruit and vegetable familiarity at T0–T4: (**a**) Number of unfamiliar fruit items; (**b**) Number of unfamiliar vegetable items. Note. All analyses were adjusted for sex, study year at T0, SES, and ethnicity. Abbreviations: HPSF, Healthy Primary School of the Future. ^★^ Significant (≤0.05) difference between full and partial HPSF after correction for baseline.

**Figure 3:**
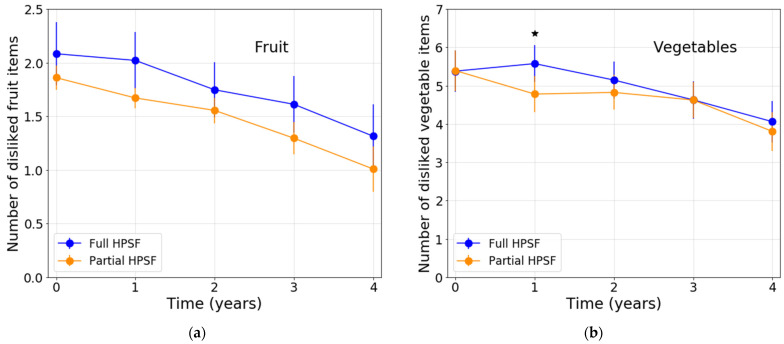
Estimated means of children’s fruit and vegetable preferences at T0–T4: (**a**) Number of disliked fruit items; (**b**) Number of disliked vegetable items. Note. All analyses were adjusted for sex, study year at T0, SES, and ethnicity. Abbreviations: HPSF, Healthy Primary School of the Future. ^★^ Significant (≤0.05) difference between full and partial HPSF after correction for baseline.

After correction for baseline, the mean number of unfamiliar vegetable items was significantly lower in full HPSFs compared with partial HPSFs at T1 and T3 (ES = −0.28 and ES = −0.35, respectively) (Table 4; Figure 2). FV preferences were largely similar in full HPSFs compared to partial HPSFs. Only at T1, a significant unfavorable intervention effect was observed, with a significant increase from baseline in the number of disliked vegetable items in full HPSFs compared to partial HPSFs (ES = 0.24) (Table 4; Figure 3). Descriptive data regarding the observed mean familiarity and preference scores for the separate FV items at T0–T4 can be found in Table S2.

### 3.3. Intervention Effects on FV Intake

After correction for baseline, the frequency of fruit intake was significantly lower in full HPSFs compared with partial HPSFs at T1 (OR = 0.609) and T4 (OR = 0.451). No significant intervention effects were observed for vegetable intake at any of the timepoints (Table 5). The effects for fruit intake could not be ascribed to a change in the percentage of participants selecting one specific response category, but were caused by a combination of changes in the various categories. Descriptive data regarding the observed mean FV intake at T0–T4 can be found in Table S3.

## 4. Discussion

The current study aimed to investigate the effects of repeated FV exposure through the full HPSF intervention on children’s FV familiarity, preferences, and intake. Although some positive intervention effects were observed for FV familiarity (mainly for vegetables), effects on preferences and intake were (almost) absent. These findings are not in line with our initial hypothesis, which described increased FV familiarity, preferences, and ultimately intake with increased exposure to FV (Figure 1). Several previous studies describing repeated exposure interventions did report positive effects on children’s familiarity, preferences, and intake [19], which raises the question of why these effects were not observed in the current study.

A possible explanation for the lack of effects on preferences and subsequently intake can be found in the form of exposure that took place. Previous research indicates that in order to be effective, exposure must take place in the same domain in which changes in preferences are desired [14]. Birch et al., for example, found that visual exposure increased visual preferences, but actual tasting was needed to increase taste preferences [23]. In the present study, FV were part of a buffet from which children composed their own lunch. This meant that children were visually exposed to FV, but taste exposure only happened after a child decided to consume FV as part of their lunch. It is likely that this increased visual exposure, together with the encouragement from teachers and educational staff, led to an increase in the number of familiar FV items, which was measured in the questionnaire. However, as FV tasting was not a standard or obligatory part of the lunch, and increased taste exposure therefore did not always happen, the increased familiarity might not have comprised an increase in taste familiarity, and an increase in taste preferences therefore remained absent. A more specific measurement of familiarity (e.g., measuring different aspects such as familiarity with the smell, texture, color, or taste of a certain FV) could have provided more insight into this possible mechanism. If the above-described phenomenon occurred, it would mean that the naturalistic nature of the intervention resulted in suboptimal effects on preferences and intake, and better results might be achieved by including a tasting component as a standard intervention component. 

Another factor that might have played a role in the limited effects is the relatively high age of the study sample. The ease of food acceptance and preference formation seems to decrease as a child matures, and more exposure over a longer time seems necessary to influence older children’s dietary habits. Moreover, preferences that are formed early in life (i.e., during a child’s first four years) tend to have a persistent long-term influence on food choices [14,47]. The current study involved children aged 7–12 years old, and the HPSF intervention might not have been powerful enough to influence the preferences already formed before this age. It would be interesting to investigate the intervention’s effects in younger children—something that was not done in the present study as the measurement instrument used in participants from study years one to three was deemed less valid due to differences in questionnaire formulation. In addition, more research into the effects of mere exposure interventions in older children would be beneficial, as studies investigating the potential of mere exposure in older age groups are relatively scarce.

It should also be noted that baseline familiarity and preferences for the various FV items measured in the present study were already high, meaning that ceiling effects might have occurred. This was most apparent for fruit items, which might explain the fact that significant intervention effects on familiarity were mostly observed for vegetables. Besides relatively high baseline familiarity and preferences, the number of exposures was higher for FV items that are generally well-known and often consumed in the Netherlands (e.g., apple, banana, tomato, cucumber). Less well-known fruit and especially vegetable items (e.g., eggplant, leek, kale) were less frequently included in the lunch cycle (Table S1). Due to this, the number of exposures might not have been sufficient to increase liking for these specific items. Increasing the number of exposures to less familiar FV items in the lunch might therefore lead to more effects on familiarity and preferences. 

From baseline to one year (T1), a significant decrease in vegetable preferences was observed in full HPSFs compared with partial HPSFs. Separate item analyses to investigate which vegetable item(s) caused this decrease in preferences were not possible due to the low variety in item responses, but experiences during the intervention might shed light on the possible causes of this negative intervention effect. In the first intervention year, several teachers and pedagogical staff reported negative experiences with the eggplant included in the lunch cycle. Children had been encouraged to try the (roasted) eggplant, but they did not like the taste of it. This could be attributed to the relatively bitter taste of eggplant, as humans and especially young children are known to have an innate aversion to bitter tastes [10]. Negative taste experiences can have a long-lasting unfavorable impact on liking of a specific food, even more so when the food has not been consumed on many other occasions [10,13]. It is therefore possible that the negative experience with eggplant during the first intervention year contributed to the decrease in vegetable preferences at T1. It could also be that teachers and pedagogical staff were too coercive in their enthusiasm to encourage children to consume FV, which negatively influenced preferences. Children might have felt forced to taste FV, while free choice with regard to FV intake is key in increasing FV preferences through repeated exposure [48]. While it is recommended to include a tasting component in the intervention to increase taste exposure, it is important that this tasting is organized in a playful, relaxed setting to minimize the potential pressure that children might feel to taste the different FV items.

At baseline, roughly 40% of participants in both groups indicated that they consumed FV on a daily basis, which is higher than the national numbers discussed in the Introduction in the present paper. A potential explanation for this could be found in the current study’s data collection procedures. As FV intake was measured using a self-report questionnaire, it is possible that children gave socially desirable responses instead of choosing responses that reflected their actual FV intake.

After correction for baseline, the frequency of fruit intake was significantly lower in full HPSFs compared with partial HPSFs after one (T1) and four years (T4). This is striking, as children in full HPSFs, as opposed to children in partial HPSFs, received a daily mid-morning snack, which was most often a fruit item. It is possible that the self-report nature of the questionnaire assessing participants’ FV intake played a role in this inconsistent result. The unfavorable effects on fruit intake might also be explained by the occurrence of compensatory behavior, which has previously been described in relation to various health behaviors such as dietary intake and PA [49,50,51,52]. Children might have consumed less fruit outside school hours to compensate for the extra fruit that they had already eaten while at school. The fact that negative effects were only observed for fruit intake and not for vegetable intake could be explained by cultural factors. In the Netherlands, fruit is often consumed as a snack, while vegetables are mostly consumed as part of the evening meal. It might therefore be easier to compensate for extra fruit intake by choosing another snack than to compensate for extra vegetable intake, as the evening meal is often consumed together as a family. Interview data from parents at full HPSFs revealed that some parents and children indeed engaged in compensatory behaviors at home following HPSF implementation—for example, by allowing their child to have a cookie instead of a healthy snack (parents) or by asking for candy instead of fruit after school because they had already eaten healthily at school (children). Inclusion of questions specifically measuring FV intake at school and at home could have provided more insight into the possible occurrence of compensatory behaviour.

Although the regular, increased exposure to FV as part of HPSF resulted in some positive effects on FV familiarity, it seems that HPSF was not successful in increasing children’s FV preferences and intake. Nevertheless, in previous studies comparing full HPSFs with control schools, favorable intervention effects on BMI z-score, waist circumference, PA, and dietary behaviors (e.g., school water consumption, vegetable and dairy intake during lunch) were found [35,36]. Mere exposure efforts combined with other school health-promoting interventions can therefore be a powerful strategy to improve children’s overall health and bring a halt to the childhood obesity epidemic. Considering the millions of children in primary schools across Europe who are currently exposed to mere exposure FV efforts by means of the freely available EU school fruit and vegetable scheme [53], it is highly relevant to further investigate under which circumstances mere exposure interventions can effectively contribute to changes in (determinants of) FV intake and eventually childhood obesity levels.

### 4.1. Post Hoc Analyses

As previously described, ceiling effects might have played a role in the limited intervention effects that were observed for FV familiarity. To further investigate this potential explanation, a post hoc analysis was performed using new summary scores for FV familiarity. Any FV item that was unfamiliar for <5% of the population at T0 was excluded from the new summary scores, resulting in the inclusion of only those FV items that showed room for improvement in familiarity at T0. This led to the exclusion of apple, banana, tangerine, pear, strawberry, and grapes from the fruit summary score, and carrot, cucumber, broccoli, tomato, and lettuce from the vegetable summary score. The results of the post hoc analysis indicate more positive intervention effects on vegetable familiarity (significant positive intervention effects at T1–T3) as compared with the primary analysis’ results (significant positive intervention effects only at T1 and T3). For fruit familiarity, the results of the post hoc analysis were comparable to the primary analysis’ results. Detailed results from the post hoc analysis can be found in Table S4. The results confirm the hypothesis that ceiling effects played a role in the limited effects that were observed for vegetable familiarity, but this influence was not clear for fruit familiarity. However, it should be noted that familiarity for fruit items was generally higher than for vegetable items (at T0, 81.8% of the fruit items were familiar for >10% of participants, while 75% of the vegetable items were familiar for >10% of participants at T0). Taking this into account, it seems that participants were already more familiar with fruit items in general, providing less room for improvement in this domain as opposed to vegetable familiarity.

As can be seen in Table S1, five of the sixteen vegetable items that were included in the questionnaire assessing subjects’ familiarity and preferences were not part of the intervention, meaning that participants were not exposed to these items between T0 and T4 as part of HPSF. It is therefore unclear whether HPSF would increase children’s familiarity with and preferences for these items. Therefore, a second post hoc analysis was performed in which the intervention’s effects on vegetable familiarity and preferences were examined after excluding these five items (Brussels sprouts, broccoli, cauliflower, green beans, and kale) from the vegetable summary scores. This post hoc analysis showed similar results for vegetable familiarity as compared with the primary analysis. However, for vegetable preferences, more positive intervention effects were observed in the post hoc analysis (significantly positive intervention effects at T1–T2). Detailed results of this post hoc analysis can be found in Table S5. The results indicate that the inclusion of the five vegetable items that were not part of HPSF in the primary analysis resulted in limited intervention effects on children’s vegetable preferences, further validating the importance of actual (taste) exposure in order to improve children’s food preferences.

### 4.2. Strengths and Limitations

Considering the study’s strengths and limitations, there are a few issues that can be noted. First, generalization of the study’s results should be done carefully, despite the relatively large sample size. Although the study population was previously found to be a good representation of the region, comparability with a national sample was moderate [34]. This was due to the lower ethnic diversity, lower SES, and higher prevalence of overweight and obesity in the sample as compared with national numbers [54]. It would therefore be beneficial to study the intervention’s effects in diverse populations. The non-randomized nature of the study can also be seen as a limitation. However, including schools based on their willingness to implement the intervention is a better reflection of the real-world process of school-based health promotion. In addition, no significant baseline differences were observed between the full and partial HPSFs, and all analyses were controlled for outcome at baseline, sex, study year at baseline, SES, and ethnicity. Due to the study design, a relatively high number of missing values could be observed for the various outcome measures. Most of the missing data occurred due to participants’ study year, as subjects were only included from study year four onwards and dropped out of the study when they left school after study year eight. This missing information was accounted for by including study year at T0 as a covariate in the analyses that were performed. Drop-out due to other reasons was observed in three subjects only in the present study.

The fact that teachers and pedagogical staff encouraged FV consumption through nudging, rewards, and positive attention might have influenced the intervention’s effects on children’s FV preferences and intake. However, such small bottom-up initiatives fit with a real-world setting, and it is likely that their effects were limited compared with those of the systematically organized lunch.

The quality of the measurement instrument used to assess FV familiarity, preferences, and intake should also be discussed. Subjective measurements, such as the questionnaire used, might lead to socially desirable answers. However, at this moment, there is no objective way to measure constructs such as food familiarity and preferences. Although the child-reported nature of the questionnaire resulted in a high response rate, it limited the amount of detail in which questions could be asked. The three-point semantic differential rating scale might not have been sensitive enough to measure FV preferences accurately. Measuring FV familiarity and preferences as continuous dimensions would have made it possible to assess slight differences more accurately. Additionally, it was not possible to assess different aspects of FV familiarity (e.g., familiarity of smell, taste, texture) with the current questionnaire. Furthermore, the outcome measures used to assess FV intake might not have captured all potential effects. It was impossible to relate changes in the familiarity and preferences of specific FV items to changes in intake, as the outcome measures that were used only assessed general FV intake and did not make a distinction between specific FV items. Furthermore, the questions measured the frequency of FV intake (in days per week) and not the amount of FV consumed. It could be that the intervention resulted in children consuming more FV on days during which they normally already consumed FV—a positive effect that could not be measured with the current outcome measures. It should also be noted that children’s FV intake outside of school is mostly regulated by parents/caregivers, who purchase and prepare the food at home. HPSF is therefore not the only factor having an impact on children’s FV intake through increasing FV exposure at school, as FV availability at home also has a large influence on children’s intake [7,55,56]. Including questions on FV availability at home and/or specifically measuring FV intake at home could have provided more insight into the influence that children’s home environments had on their FV intake in the present study.

A limitation related to data analysis might be the fact that although multiple testing was performed, no correction was applied. However, it is unlikely that correction would have changed our overall conclusion that effects on preferences and intake were (almost) absent, as *p*-values for the various outcomes would remain largely non-significant. A final limitation is related to the HPSF intervention in general. As recommended by various researchers in the field, it seems that interventions aimed at improving children’s lifestyle habits should target all environments in which the child is involved (e.g., school, home, sports associations) [57,58,59]. The main focus of HPSF was the school environment, but more positive results might be observed when parents and the home setting in general are more explicitly included in the intervention as well. 

## 5. Conclusions

The HPSF intervention showed limited effects on FV familiarity, and effects on FV preferences and intake were (almost) absent. These findings are not in line with our initial hypothesis, which described increased FV familiarity, preferences, and ultimately intake with regular, increased exposure to FV as part of the HPSF intervention.

The lack of effects might be explained by the presence of a ceiling effect, the relatively high age of the included sample, and the fact that actual taste exposure as part of the intervention was limited. Although the HPSF intervention was previously found to have positive effects on various health outcomes in children, it can be concluded that the intervention was not powerful enough to influence children’s FV preferences and intake. Considering the widespread implementation of school-based mere exposure efforts (e.g., by means of the EU school fruit and vegetable scheme), it is highly relevant to further investigate under which circumstances these efforts can effectively contribute to changes in (determinants of) FV intake and eventually reduce childhood obesity levels.

## Figures and Tables

**Table 1:** Participants included in the present study, specified for T0–T4.

	Number of Included Participants, Divided Per Study Year *(Study Year at T0 in Brackets)*									
	Study Year 4		Study Year 5		Study Year 6		Study Year 7		Study Year 8 ^1^	
	Full HPSF	Partial HPSF	Full HPSF	Partial HPSF	Full HPSF	Partial HPSF	Full HPSF	Partial HPSF	Full HPSF	Partial HPSF
**Timepoint**										
T0 (2015)	48	53	53	61	51	45	58	67		
T1 (2016)	51 (3)	59 (3)	50 (4)	56 (4)	57 (5)	62 (5)	53 (6)	45 (6)	60 (7)	67 (7)
T2 (2017)	60 (2)	80 (2)	52 (3)	64 (3)	55 (4)	59 (4)	59 (5)	62 (5)	53 (6)	47 (6)
T3 (2018)	48 (1)	49 (1)	60 (2)	80 (2)	52 (3)	64 (3)	55 (4)	59 (4)	59 (5)	62 (5)
T4 (2019)	3 (1) ^2^	6 (1) ^2^	48 (1)	52 (1)	63 (2)	82 (2)	53 (3)	62 (3)	56 (4)	60 (4)

^1^ At T0, no participants from study year eight were included as no data could be collected from these subjects at later measurement points (T1–T4) because they had left school. ^2^ Participants repeated study year one.

**Table 2:** Fruit and vegetable items assessed in the questionnaire.

Fruit	Vegetables
Apple	Cucumber
Banana	Tomato
Grapes	Carrot
Kiwi	Bell pepper
Mango	Lettuce
Tangerine	Zucchini
Pear	Spinach
Orange	Eggplant
Melon	Onion
Pineapple	Leek
Peach	Peas
Strawberries	Brussels sprouts
	Broccoli
	Green beans
	Cauliflower
	Kale

**Table 3:** Characteristics of participants at baseline (T0).

	Total (*n* = 833)				Full HPSF (*n* = 394) With Exposure to FV				Partial HPSF (*n* = 439) Without Exposure to FV					
	*n*	% Missing Values	%/M	SD	*n*	% Missing Values	%/M	SD	*n*	% Missing Values	%/M	SD	X^2^/t-Value	*p*
Sex (% boys) ^1^	833	0	48		394	0	47		439	0	49		0.267	0.605
Age (years)	833	0	7.5	2.2	394	0	7.5	2.1	439	0	7.5	2.2	0.534	0.593
Ethnicity (% Western) ^1^	599	28	96		287	27	94		312	29	97		2.566	0.109
SES (%) ^1^ Lowest tertile	629 189	25	30		292 82	26	28		337 107	23	32		1.180	0.554
Middle tertile	233		37		109		37		124		37			
Highest tertile	207		33		101		35		106		32			
Familiarity (mean *n* unfamiliar items) ^2^ Fruit (range 0–12)	418	50	0.9	1.6	198	50	1.0	1.6	220	50	0.9	1.5	0.155	0.877
Vegetables (range 0–16)	420	50	1.8	2.2	199	50	1.8	2.1	221	50	1.7	2.3	0.231	0.818
Preference (mean *n* disliked items) ^2^ Fruit (range 0–12)	393	53	1.4	1.8	184	53	1.5	1.9	209	52	1.3	1.7	0.982	0.327
Vegetables (range 0–16)	376	55	4.1	3.1	177	55	4.1	3.1	199	55	4.1	3.2	−0.235	0.814
Fruit intake (%) ^1^ (almost) Never	403 21	52	5		190 11	52	6		213 10	52	5		3.684	0.298
Sometimes (1–3 days per week)	82		20		31		16		51		24			
Often (4–6 days per week)	125		31		62		33		63		30			
Every day	175		43		86		45		89		42			
Vegetable intake (%) ^1^ (almost) Never	380 15	54	4		184 8	53	4		196 7	55	4		5.026	0.170
Sometimes (1–3 days per week)	62		16		22		12		40		20			
Often (4–6 days per week)	143		38		72		39		71		36			
Every day	160		42		82		45		78		40			

Note. All children who were exposed to the school environment at T0, participated in ≥1 measurement from T1 to T4 while in study years four to eight, and had a valid response for ≥1 outcome during these measurementsare included in the present table. For participants who had not yet enrolled in the study at T0, demographic characteristics from the first available measurement are included (age at T0 was calculated using age at participants’ first available measurement). Due to the dynamic open cohort and the fact that only data collected in study years four to eight are included in the current study, baseline FV familiarity, preference, and intake scores are not available for all included participants. This is because some subjects were not yet enrolled in the study at T0, or participants were not yet in study years four to eight at T0, meaning that no baseline data were collected for these subjects. This can be observed in the table by the relatively high amount of missing values for these outcomes. Note. Due to the non-randomized nature of the study, baseline differences between the full and partial HPSF were investigated. Abbreviations: HPSF, Healthy Primary School of the Future; FV, fruit and vegetables; M, mean; SD, standard deviation; SES, socioeconomic status. ^1^ Analyzed by X^2^ test. ^2^ Calculated for participants with a maximum missing rate of 25% (corresponding to ≤3 missing fruit items or ≤4 missing vegetable items).

**Table 4:** Estimated intervention effects on fruit and vegetable familiarity and preferences.

			Full HPSF vs. Partial HPSF		
			B (95% CI)	*p*	ES
**Familiarity**	**Number of unfamiliar fruit items**	**T0–T1**	−0.222 (−0.487; −0.043)	0.101	−0.16
		**T0–T2**	0.031 (−0.271; 0.332)	0.841	0.02
		**T0–T3**	−0.102 (−0.426; 0.223)	0.538	−0.07
		**T0–T4**	0.351 (−0.005; 0.708)	0.053	0.25
	**Number of unfamiliar vegetable items**	**T0–T1**	−0.604 (−1.058; −0.150)	0.009 *	−0.28
		**T0–T2**	−0.465 (−0.990; 0.060)	0.083	−0.21
		**T0–T3**	−0.768 (−1.333; −0.203)	0.008 *	−0.35
		**T0–T4**	−0.469 (−1.083; 0.146)	0.135	−0.22
**Preferences**	**Number of disliked fruit items**	**T0–T1**	0.127 (−0.203; 0.457)	0.450	0.07
		**T0–T2**	−0.031 (−0.423; 0.361)	0.877	−0.02
		**T0–T3**	0.094 (−0.335; 0.523)	0.667	0.05
		**T0–T4**	0.083 (−0.391; 0.558)	0.730	0.04
	**Number of disliked vegetable items**	**T0–T1**	0.808 (0.151; 1.464)	0.016 *	0.24
		**T0–T2**	0.336 (−0.431; 1.103)	0.390	0.10
		**T0–T3**	0.016 (−0.821; 0.853)	0.970	0.00
		**T0–T4**	0.266 (−0.651; 1.184)	0.569	0.08

Note. Analyzed by linear mixed model analyses. All analyses were adjusted for sex, study year at T0, SES, and ethnicity. Abbreviations: HPSF, Healthy Primary School of the Future; CI, confidence interval; ES, effect size. * Significant (≤0.05) difference between full and partial HPSF.

**Table 5:** Estimated intervention effects on fruit and vegetable intake.

		Full HPSF vs. Partial HPSF	
		OR (95% CI)	*p*
**Fruit intake**	**T0–T1**	0.609 (0.389; 0.952)	0.030 *
	**T0–T2**	0.626 (0.383; 1.021)	0.061
	**T0–T3**	0.798 (0.478; 1.334)	0.390
	**T0–T4**	0.451 (0.259; 0.786)	0.005 *
**Vegetable intake**	**T0–T1**	0.828 (0.490; 1.402)	0.483
	**T0–T2**	1.330 (0.750; 2.359)	0.329
	**T0–T3**	1.009 (0.548; 1.856)	0.978
	**T0–T4**	1.008 (0.538; 1.887)	0.981

Note. Fruit and vegetable intake was coded as follows: 1 = (almost) never, 2 = sometimes, 3 = often, 4 = every day. Analyzed by ordinal logistic model analyses. All analyses were adjusted for sex, study year at T0, SES, and ethnicity. Abbreviations: HPFS, Healthy Primary School of the Future; OR, odds ratio; CI, confidence interval. * Significant (≤0.05) difference between full and partial HPSF.

## Data Availability

Data supporting the study’s findings were collected as part of the “Healthy Primary School of the Future” quasi-experimental study. All individual participant data that underlie the results reported in this article (text, tables, figures, and appendices) will be available after de-identification, including the statistical analysis plan. Data will be available with suitable qualified researchers beginning nine months and ending ten years following article publication on the four-year effects on the primary outcome measure of the original study (i.e., BMI z-score) as registered in the ClinicalTrials.gov database NCT02800616. Data will only be shared with parties who provide a methodologically sound proposal, or for individual participant data meta-analysis. Proposals should be directed to mth.hahnraths@maastrichtuniversity.nl. To gain access, data requestors will need to sign a data access agreement. Three years following article publication on the four-year effects on the primary outcome measure of the original study (i.e., BMI z-score), data will be available in our university data warehouse but without investigator support other than deposited metadata.

## Supplementary Materials

The following are available online at https://www.mdpi.com/article/10.3390/nu13093241/s1, Table S1: Frequency of exposure between T0 and T4 per fruit and vegetable item, Figure S1: Trial profile, Table S2: Observed mean fruit and vegetable familiarity and preference scores at the various time at the various time points (T0–T4), Table S3: Observed mean fruit and vegetable intake at the various time points (T0–T4), Table S4: Estimated intervention effects on fruit and vegetable familiarity as examined by a post hoc analysis using new summary scores, Table S5: Estimated intervention effects on vegetable familiarity and preferences as examined by a post hoc analysis.
