# Supplementary material for: The Effects of the Healthy Primary School of the Future on Children’s Fruit and Vegetable Preferences, Familiarity and Intake
Source: Nutrients. 2021 Sep 17;13(9):3241. doi: 10.3390/nu13093241 (PMC8469863; doi:10.3390/nu13093241)
Supplement: Supplementary file 1 [file nutrients-13-03241-s001.zip › Figure S1. Trial profile..pdf]

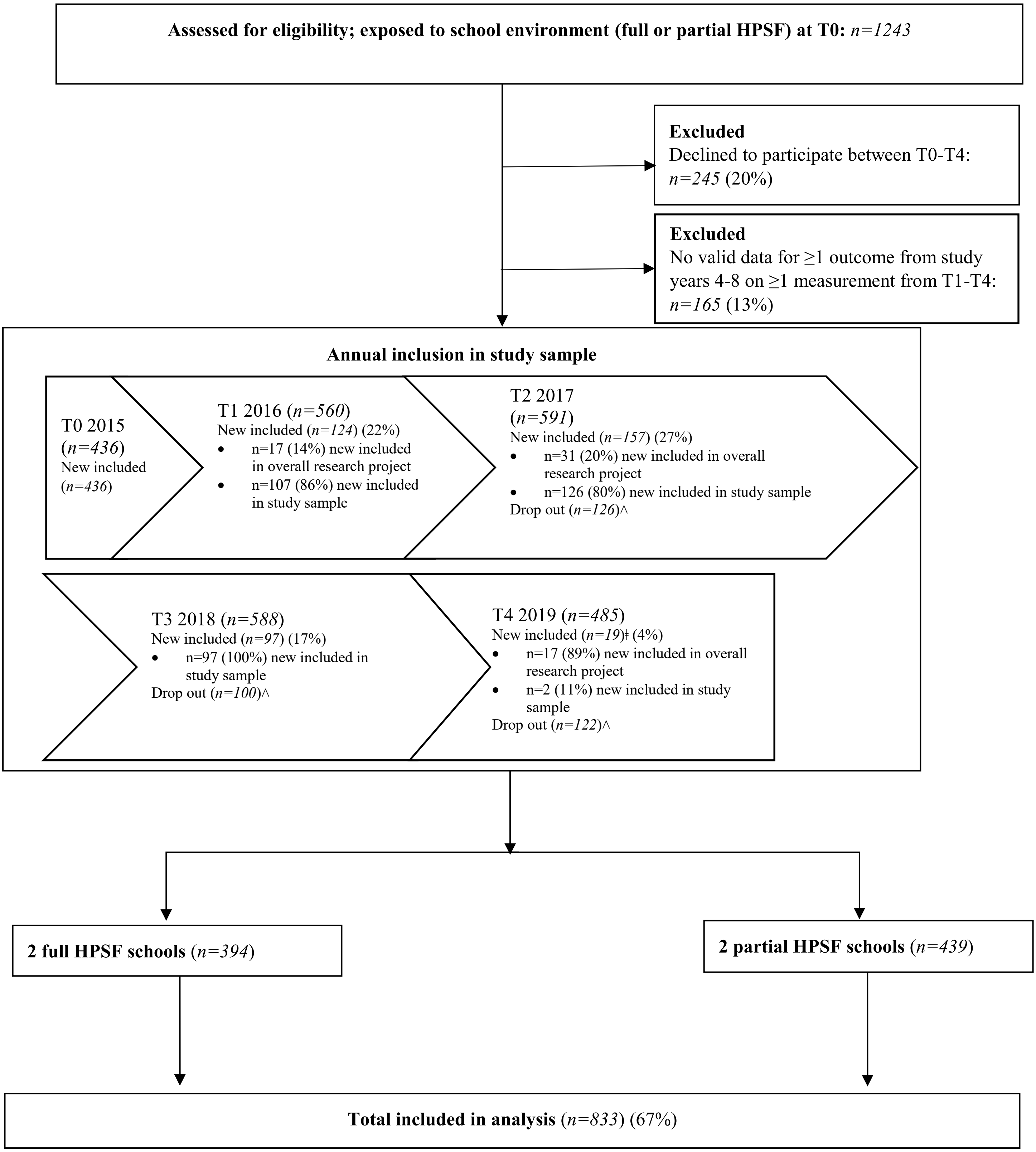

Figure S1. Trial profile.

Abbreviations: HPSF, Healthy Primary School of the Future.

ǂ Newly included participants include subjects who started participation in the overall research project after T0 and subjects who already participated in the research project while in study years 1-3, but are included in the present study's sample from the moment they are in study years 4-8.

^ Reasons for drop out over the four years include graduation (n=345), or other (n=3) including migration, actively stopping participation or switching to other included schools.

Note. Between T0-T1, no drop out was observed as subjects were only included in the present study if they had ≥1 valid measurement between T1-T4. Reported drop out numbers only include subjects dropping out of the current study, not the overall research project.
