# Supplementary material for: The Effects of the Healthy Primary School of the Future on Children’s Fruit and Vegetable Preferences, Familiarity and Intake
Source: Nutrients. 2021 Sep 17;13(9):3241. doi: 10.3390/nu13093241 (PMC8469863; doi:10.3390/nu13093241)
Supplement: Supplementary file 1 [file nutrients-13-03241-s001.zip › Table S1. Frequency of exposure between T0 and T4 per fruit and vegetable item..pdf]

Table S1. Frequency of exposure between T0 and T4 per fruit and vegetable item.

Fruit	Frequency of exposure†	Vegetables	Frequency of exposure†
Apple [1,‡]	476	Cucumber‡	355
Banana‡	263	Tomato [6,‡]	351
Grapes‡	189	Carrot‡	349
Kiwi [2,‡]	126	Bell pepper [7,‡]	226
Mango [3,‡]	115	Lettuce [8,‡]	193
Tangerine‡	104	Mixed salad	122
Pear‡	100	Baby corn	97
Orange‡	90	Beet	68
Melon [4,‡]	84	Avocado	63
Pineapple‡	65	Radish	58
Fruit salad	60	Celeriac	50
Nectarine	32	Cress	47
Mineola	21	Zucchini [9,‡]	44
Plums	20	Celery	44
Peach‡	16	Spinach‡	30
Apricot	11	Pickle	28
Strawberries‡	9	Sweet potato	21
Grapefruit [5]	7	Seaweed	19
Pomegranate	7	Eggplant [10,‡]	10
Blackberries	7	Fennel	10
Blueberries	3	Cabbage [11]	8
Kaki fruit	3	Pumpkin	7
Dragon fruit	3	Onion‡	6
Passion fruit	3	Leek‡	6
Carambola	3	Rhubarb	3
Papaya	3	Peas‡	1.5
		Asparagus	1.5
		Brussels sprouts‡	0
		Broccoli‡	0
		Green beans‡	0
		Cauliflower‡	0
		Kale‡	0

[1] Including dried apple; [2] Including hardy kiwi; [3] Including dried mango; [4] Including watermelon, cantaloupe, honeydew; [5] Including pomelo; [6] Including snack tomatoes; [7] Including grilled bell pepper; [8] Including arugula, crop, lamb's lettuce, iceberg lettuce, little gem, winter purslane; [9] Including grilled zucchini; [10] Including grilled eggplant; [11] Including red cabbage, bok choy.

†Amount of exposures corresponds to the number of times the FV items were included in the lunch cycle. Amount of exposures includes half portions (as the lunch cycle prescribed that the caterer could choose between two FV types, or a mix of both FV types was presented).

‡Fruit and vegetable types evaluated in the current study.
