# Supplementary material for: The Effects of the Healthy Primary School of the Future on Children’s Fruit and Vegetable Preferences, Familiarity and Intake
Source: Nutrients. 2021 Sep 17;13(9):3241. doi: 10.3390/nu13093241 (PMC8469863; doi:10.3390/nu13093241)
Supplement: Supplementary file 1 [file nutrients-13-03241-s001.zip › Table S2. Observed mean fruit and vegetable familiarity and preference scores at the various time points (T0-T4)..pdf]

Table S2. Observed mean fruit and vegetable familiarity and preference scores at the various time points (T0-T4).

T0

**Item**	**Full HPSF**						**Partial HPSF**					
	**Familiarity**		**Liking**				**Familiarity**		**Liking**			
	n	% (n) Unfamiliar	n	% (n) Dislike	% (n) Neutral	% (n) Like	n	% (n) Unfamiliar	n	% (n) Dislike	% (n) Neutral	% (n) Like
Apple	198	1 (1)	197	2 (4)	21 (42)	77 (151)	219	0 (0)	219	3 (6)	16 (35)	81 (178)
Banana	198	0 (0)	198	16 (31)	31 (62)	53 (105)	219	2 (4)	215	9 (19)	18 (39)	73 (157)
Tangerine	198	4 (8)	190	15 (28)	34 (64)	52 (98)	220	2 (5)	215	14 (30)	28 (60)	58 (125)
Orange	197	7 (14)	183	19 (34)	28 (51)	54 (98)	219	5 (11)	208	17 (36)	25 (52)	58 (120)
Pineapple	197	18 (35) [1]	162	20 (32)	22 (35)	59 (95)	220	15 (32) [1]	188	22 (42)	19 (36)	59 (110)
Pear	197	3 (6)	190	12 (22)	34 (64)	55 (104)	219	1 (2)	217	18 (39)	18 (40)	64 (138)
Melon	197	5 (10)	187	11 (21)	9 (16)	80 (150)	219	6 (13)	206	6 (13)	8 (16)	86 (177)
Kiwi	198	7 (13)	185	21 (38)	22 (40)	58 (107)	218	6 (13)	205	14 (28)	16 (33)	70 (144)
Strawberry	198	2 (4)	193	3 (6)	10 (20)	87 (167)	220	1 (3)	217	3 (6)	3 (6)	95 (205)
Mango	198	32 (64) [1]	133	21 (28)	23 (31)	56 (74)	221	36 (79) [1]	142	21 (30)	18 (26)	61 (86)
Peach	198	15 (29) [1]	169	12 (20)	27 (45)	62 (104)	221	16 (36) [1]	185	10 (18)	16 (29)	75 (138)
Grapes	198	2 (4)	194	6 (12)	18 (35)	76 (147)	221	2 (5)	216	2 (4)	11 (24)	87 (188)
Cauliflower	198	6 (12)	186	20 (37)	28 (52)	52 (97)	222	5 (10)	212	21 (44)	26 (55)	53 (113)
Zucchini	198	27 (53) [1]	144	56 (80)	22 (31)	23 (33) [2]	219	27 (59) [1]	160	50 (80)	16 (26)	34 (54) [2]
Carrot	198	0 (0)	198	11 (22)	24 (48)	65 (128)	222	1 (2)	220	16 (35)	17 (37)	67 (148)
Cucumber	199	1 (1)	198	4 (8)	11 (21)	85 (169)	222	1 (3)	219	4 (9)	10 (22)	86 (188)
Bell pepper	199	4 (7)	192	23 (44)	17 (32)	60 (116)	222	8 (17)	205	22 (44)	20 (41)	56 (120)
Broccoli	199	3 (5)	193	18 (35)	25 (48)	57 (110)	222	5 (11)	211	20 (42)	23 (48)	57 (121)
Green beans	198	7 (13)	185	21 (39)	28 (51)	51 (95)	221	6 (13)	208	28 (59)	23 (48)	49 (101) [2]
Eggplant	198	54 (107) [1]	91	56 (51)	24 (22)	20 (18) [2]	221	46 (101) [1]	120	55 (66)	23 (27)	23 (27) [2]
Kale	197	25 (49) [1]	148	26 (38)	22 (32)	53 (78)	222	19 (42) [1]	180	26 (47)	21 (38)	53 (95)
Brussels sprouts	199	9 (18)	181	54 (97)	17 (30)	30 (54) [2]	221	6 (13)	208	50 (103)	12 (25)	39 (80) [2]
Leek	195	15 (29) [1]	166	36 (60)	33 (55)	31 (51) [2]	216	17 (36) [1]	180	32 (58)	32 (58)	36 (64) [2]
Tomato	199	2 (4)	195	28 (55)	16 (32)	55 (108)	219	3 (6)	213	24 (52)	13 (27)	63 (134)
Lettuce	198	3 (6)	192	10 (20)	16 (30)	74 (142)	220	5 (11)	209	10 (21)	17 (36)	73 (152)
Spinach	198	5 (10)	188	25 (46)	18 (34)	57 (108)	220	7 (16)	204	25 (50)	19 (39)	56 (115)
Onion	198	8 (15)	183	35 (64)	32 (59)	33 (60) [2]	216	6 (13)	203	44 (90)	25 (50)	31 (63) [2]
Peas	199	13 (26) [1]	173	24 (42)	21 (37)	54 (94)	220	15 (33) [1]	187	25 (47)	24 (44)	51 (96)

T1

Item	Full HPSF						Partial HPSF					
	Familiarity		Liking				Familiarity		Liking			
	n	% (n) Unfamiliar	n	% (n) Dislike	% (n) Neutral	% (n) Like	n	% (n) Unfamiliar	n	% (n) Dislike	% (n) Neutral	% (n) Like
Apple	263	0 (0)	263	3 (9)	24 (64)	72 (190)	283	0 (1)	282	2 (6)	17 (48)	81 (228)
Banana	263	0 (0)	263	15 (39)	23 (60)	62 (164)	283	0 (1)	282	9 (25)	26 (73)	65 (184)
Tangerine	262	3 (7)	255	18 (46)	33 (84)	49 (125) [2]	283	4 (11)	272	14 (37)	26 (71)	60 (164)
Orange	261	8 (20)	241	21 (50)	34 (83)	45 (108) [2]	282	5 (14)	268	9 (25)	28 (74)	63 (169)
Pineapple	262	11 (29) [1]	232	21 (49)	17 (40)	62 (143)	282	16 (45) [1]	236	20 (47)	19 (45)	61 (144)
Pear	261	0 (1)	260	15 (40)	31 (81)	54 (139)	282	2 (6)	276	14 (39)	20 (55)	66 (182)
Melon	263	1 (3)	260	14 (36)	12 (32)	74 (192)	282	4 (10)	271	7 (20)	7 (20)	85 (231)
Kiwi	263	4 (11)	252	20 (50)	26 (65)	54 (137)	281	8 (22)	259	13 (33)	22 (57)	65 (169)
Strawberry	263	2 (5)	258	5 (12)	7 (18)	88 (228)	283	1 (4)	279	3 (9)	5 (13)	92 (257)
Mango	261	25 (65) [1]	196	30 (58)	20 (40)	50 (98) [2]	283	36 (102) [1]	181	18 (32)	19 (34)	64 (115)
Peach	263	15 (39) [1]	224	18 (40)	18 (41)	64 (143)	282	14 (39) [1]	243	12 (29)	16 (38)	72 (176)
Grapes	263	2 (4)	259	4 (10)	18 (46)	78 (203)	282	1 (4)	278	4 (11)	11 (31)	85 (236)
Cauliflower	264	5 (12)	252	20 (50)	35 (87)	46 (115) [2]	283	4 (10)	273	20 (54)	30 (81)	51 (138)
Zucchini	264	14 (36) [1]	227	58 (131)	20 (45)	23 (51) [2]	282	23 (64) [1]	217	48 (105)	25 (55)	26 (57) [2]
Carrot	264	1 (2)	262	16 (41)	23 (60)	62 (161)	282	1 (3)	279	13 (37)	20 (56)	67 (186)
Cucumber	263	1 (2)	260	5 (13)	6 (16)	89 (231)	283	1 (4)	279	4 (12)	8 (21)	88 (246)
Bell pepper	263	3 (8)	255	23 (59)	18 (46)	59 (150)	283	5 (13)	270	16 (44)	16 (44)	67 (182)
Broccoli	264	1 (2)	261	21 (55)	23 (61)	56 (145)	282	5 (13)	269	20 (55)	18 (49)	61 (165)
Green beans	264	3 (8)	256	25 (63)	35 (89)	41 (104) [2]	280	8 (21)	259	25 (65)	27 (71)	48 (123) [2]
Eggplant	262	34 (88) [1]	174	71 (123)	19 (33)	10 (18) [2]	280	50 (139) [1]	141	53 (75)	23 (32)	24 (34) [2]
Kale	263	24 (64) [1]	199	34 (67)	26 (51)	41 (81) [2]	282	22 (61) [1]	220	24 (52)	20 (44)	56 (124)
Brussels sprouts	261	7 (19)	242	53 (128)	18 (44)	29 (70) [2]	282	9 (25)	257	50 (129)	18 (45)	32 (83) [2]
Leek	261	12 (31) [1]	230	41 (94)	35 (81)	24 (55) [2]	279	18 (50) [1]	229	28 (65)	31 (70)	41 (94) [2]
Tomato	262	2 (4)	257	28 (71)	16 (41)	56 (145)	282	3 (9)	273	25 (67)	15 (42)	60 (164)
Lettuce	262	2 (4)	258	16 (42)	27 (69)	57 (147)	281	5 (13)	268	8 (21)	18 (47)	75 (200)
Spinach	262	5 (14)	247	25 (61)	19 (47)	56 (139)	282	9 (24)	258	21 (53)	17 (43)	63 (162)
Onion	262	6 (15)	247	40 (98)	36 (89)	24 (60) [2]	282	8 (23)	259	32 (83)	35 (90)	33 (86) [2]
Peas	260	8 (20)	239	25 (60)	29 (70)	46 (109) [2]	282	10 (28) [1]	254	21 (52)	29 (73)	51 (129)

T2

Item	Full HPSF						Partial HPSF					
	Familiarity		Liking				Familiarity		Liking			
	n	% (n) Unfamiliar	n	% (n) Dislike	% (n) Neutral	% (n) Like	n	% (n) Unfamiliar	n	% (n) Dislike	% (n) Neutral	% (n) Like
Apple	254	0 (0)	254	2 (6)	25 (63)	73 (185)	286	1 (2)	284	4 (12)	19 (53)	77 (219)
Banana	254	0 (0)	254	13 (33)	23 (59)	64 (162)	286	1 (2)	284	11 (32)	23 (65)	66 (187)
Tangerine	252	4 (10)	242	17 (40)	31 (76)	52 (126)	286	2 (5)	281	17 (47)	29 (80)	55 (154)
Orange	252	9 (23)	229	21 (47)	31 (71)	49 (111) [2]	284	6 (18)	265	14 (37)	31 (82)	55 (146)
Pineapple	250	14 (34) [1]	216	20 (44)	22 (48)	57 (124)	285	18 (51) [1]	234	21 (48)	21 (48)	59 (138)
Pear	253	0 (1)	252	12 (31)	29 (73)	59 (148)	285	3 (7)	278	17 (46)	23 (65)	60 (167)
Melon	253	3 (7)	246	11 (26)	12 (29)	78 (191)	285	3 (8)	277	8 (23)	8 (22)	84 (232)
Kiwi	252	5 (12)	240	21 (51)	28 (66)	51 (123)	285	7 (20)	265	18 (47)	22 (59)	60 (159)
Strawberry	254	1 (2)	252	5 (12)	9 (23)	86 (217)	286	1 (4)	282	4 (12)	5 (15)	90 (255)
Mango	254	22 (56) [1]	198	35 (70)	21 (41)	44 (87) [2]	286	38 (108) [1]	178	22 (39)	15 (27)	63 (112)
Peach	253	14 (36) [1]	217	21 (45)	21 (45)	59 (127)	286	19 (54) [1]	232	19 (44)	20 (46)	61 (142)
Grapes	253	1 (3)	250	8 (19)	17 (43)	75 (188)	285	2 (5)	280	4 (11)	15 (43)	81 (226)
Cauliflower	254	3 (7)	247	23 (57)	31 (76)	46 (114) [2]	285	5 (13)	272	28 (77)	27 (72)	45 (123) [2]
Zucchini	254	10 (25) [1]	229	66 (150)	21 (49)	13 (30) [2]	284	25 (70) [1]	214	52 (111)	25 (54)	23 (49) [2]
Carrot	253	0 (1)	252	14 (36)	27 (69)	58 (147)	283	1 (2)	281	18 (50)	23 (65)	59 (166)
Cucumber	252	1 (2)	250	7 (18)	10 (24)	83 (208)	284	1 (2)	282	4 (12)	8 (22)	88 (248)
Bell pepper	254	2 (6)	248	29 (71)	19 (48)	52 (129)	284	5 (15)	269	19 (52)	16 (44)	64 (173)
Broccoli	254	2 (5)	249	21 (53)	27 (67)	52 (129)	286	3 (9)	277	23 (63)	23 (63)	55 (151)
Green beans	253	6 (15)	238	26 (61)	29 (68)	46 (109) [2]	281	6 (16)	265	29 (77)	25 (65)	46 (123) [2]
Eggplant	253	39 (98) [1]	155	74 (114)	18 (28)	8 (13) [2]	284	56 (160) [1]	124	67 (83)	20 (25)	13 (16) [2]
Kale	251	20 (50) [1]	201	35 (70)	25 (50)	40 (81) [2]	286	24 (69) [1]	217	29 (63)	26 (57)	45 (97) [2]
Brussels sprouts	251	6 (15)	236	51 (121)	18 (43)	31 (72) [2]	285	7 (21)	264	55 (145)	15 (39)	30 (80) [2]
Leek	249	12 (29) [1]	220	40 (87)	32 (70)	29 (63) [2]	282	21 (60) [1]	222	38 (84)	30 (66)	32 (72) [2]
Tomato	253	2 (5)	248	32 (79)	11 (27)	57 (142)	284	3 (9)	275	28 (77)	15 (42)	57 (156)
Lettuce	253	4 (9)	244	15 (36)	25 (62)	60 (146)	285	4 (12)	273	12 (33)	22 (61)	66 (179)
Spinach	253	5 (13)	240	28 (67)	19 (45)	53 (128)	285	7 (21)	264	30 (78)	17 (45)	53 (141)
Onion	252	6 (16)	236	43 (102)	31 (72)	26 (62) [2]	284	7 (21)	263	43 (112)	27 (72)	30 (79) [2]
Peas	252	8 (19)	233	26 (60)	23 (54)	51 (119)	285	11 (32) [1]	253	31 (78)	26 (65)	44 (110) [2]

T3

Item	Full HPSF						Partial HPSF					
	Familiarity		Liking				Familiarity		Liking			
	n	% (n) Unfamiliar	n	% (n) Dislike	% (n) Neutral	% (n) Like	n	% (n) Unfamiliar	n	% (n) Dislike	% (n) Neutral	% (n) Like
Apple	246	0 (0)	246	1 (3)	23 (57)	76 (186)	289	0 (0)	289	5 (13)	16 (47)	79 (229)
Banana	246	0 (0)	246	12 (29)	24 (60)	64 (157)	289	0 (0)	289	10 (30)	18 (52)	72 (207)
Tangerine	245	2 (5)	240	14 (33)	31 (74)	55 (133)	289	1 (4)	285	12 (35)	25 (72)	63 (178)
Orange	246	5 (12)	233	23 (54)	27 (63)	50 (116) [2]	289	4 (10)	278	10 (29)	27 (75)	63 (174)
Pineapple	246	12 (29) [1]	217	26 (56)	20 (44)	54 (117)	289	10 (28) [1]	261	22 (56)	14 (37)	64 (168)
Pear	246	1 (2)	244	16 (39)	28 (67)	57 (138)	288	1 (3)	285	17 (48)	25 (72)	58 (165)
Melon	246	4 (9)	237	13 (30)	6 (15)	81 (192)	286	1 (4)	282	9 (24)	9 (24)	83 (234)
Kiwi	246	4 (9)	236	21 (49)	22 (52)	57 (135)	288	6 (18)	270	15 (41)	21 (56)	64 (173)
Strawberry	245	1 (3)	242	3 (7)	8 (19)	89 (216)	289	1 (2)	287	6 (18)	4 (12)	90 (257)
Mango	245	21 (52) [1]	193	33 (64)	25 (49)	42 (80) [2]	287	30 (87) [1]	200	19 (38)	19 (37)	63 (125)
Peach	247	13 (33) [1]	214	23 (50)	25 (53)	52 (111)	287	20 (56) [1]	231	15 (35)	20 (45)	65 (151)
Grapes	246	0 (1)	245	7 (17)	17 (42)	76 (186)	287	2 (5)	282	4 (12)	14 (39)	82 (231)
Cauliflower	247	5 (11)	236	23 (55)	24 (56)	53 (125)	287	5 (14)	273	26 (71)	29 (79)	45 (123) [2]
Zucchini	245	11 (27) [1]	218	65 (142)	18 (40)	17 (36) [2]	287	25 (71) [1]	216	51 (111)	27 (59)	21 (46) [2]
Carrot	247	0 (1)	246	17 (41)	22 (54)	61 (151)	286	2 (7)	279	17 (48)	23 (64)	60 (167)
Cucumber	247	1 (2)	245	7 (16)	12 (29)	82 (200)	285	1 (2)	283	6 (16)	6 (17)	88 (250)
Bell pepper	246	4 (9)	237	25 (60)	13 (30)	62 (147)	287	4 (12)	275	22 (61)	18 (49)	60 (165)
Broccoli	246	2 (5)	241	25 (61)	21 (51)	54 (129)	285	3 (8)	276	21 (59)	26 (72)	53 (145)
Green beans	245	5 (13)	232	27 (62)	27 (62)	47 (108) [2]	286	7 (19)	267	29 (76)	29 (77)	43 (114) [2]
Eggplant	244	39 (96) [1]	148	72 (107)	17 (25)	11 (16) [2]	286	53 (152) [1]	134	67 (90)	18 (24)	15 (20) [2]
Kale	247	22 (54) [1]	193	33 (64)	27 (52)	40 (77) [2]	287	23 (67) [1]	220	33 (72)	27 (60)	40 (88) [2]
Brussels sprouts	241	6 (14)	227	52 (117)	18 (40)	31 (70) [2]	290	9 (27)	263	54 (141)	14 (37)	32 (85) [2]
Leek	241	16 (38) [1]	203	41 (84)	29 (59)	30 (60) [2]	290	25 (71) [1]	219	42 (92)	29 (64)	29 (63) [2]
Tomato	241	2 (4)	237	27 (65)	16 (37)	57 (135)	289	4 (10)	279	28 (78)	13 (37)	59 (164)
Lettuce	242	2 (4)	238	18 (43)	24 (56)	58 (139)	289	4 (11)	278	16 (43)	23 (63)	62 (172)
Spinach	242	5 (12)	230	25 (58)	17 (40)	57 (132)	290	11 (31) [1]	259	29 (75)	19 (50)	52 (134)
Onion	242	4 (9)	232	44 (101)	32 (73)	25 (58) [2]	290	9 (27)	263	41 (108)	25 (65)	34 (90) [2]
Peas	241	10 (23) [1]	218	27 (59)	24 (52)	49 (107) [2]	290	14 (41) [1]	249	32 (80)	25 (63)	43 (106) [2]

T4												
**Item**	**Full HPSF**						**Partial HPSF**					
	**Familiarity**		**Liking**				**Familiarity**		**Liking**			
	n	% (n) Unfamiliar	n	% (n) Dislike	% (n) Neutral	% (n) Like	n	% (n) Unfamiliar	n	% (n) Dislike	% (n) Neutral	% (n) Like
Apple	192	0 (0)	192	2 (3)	21 (40)	78 (149)	239	0 (0)	239	4 (10)	18 (44)	77 (185)
Banana	192	0 (0)	192	14 (27)	26 (49)	60 (116)	239	0 (0)	239	9 (22)	22 (52)	69 (165)
Tangerine	94	5 (5)	187	13 (24)	35 (65)	52 (98)	98	2 (2)	236	10 (23)	31 (72)	60 (141)
Orange	192	7 (14)	178	15 (27)	35 (62)	50 (89) [2]	237	2 (5)	232	11 (25)	24 (56)	65 (151)
Pineapple	191	16 (31) [1]	160	26 (41)	17 (27)	58 (92)	238	5 (12)	226	19 (43)	20 (44)	62 (139)
Pear	191	1 (1)	190	17 (33)	32 (60)	51 (97)	239	0 (0)	239	16 (37)	26 (62)	59 (140)
Melon	192	4 (8)	184	11 (20)	7 (13)	82 (151)	239	0 (1)	238	7 (16)	8 (19)	85 (203)
Kiwi	192	4 (8)	184	18 (33)	26 (48)	56 (103)	239	4 (9)	230	19 (44)	20 (46)	61 (140)
Strawberry	192	1 (2)	190	1 (2)	4 (8)	95 (180)	239	0 (1)	238	4 (10)	6 (13)	90 (215)
Mango	193	21 (41) [1]	152	32 (49)	16 (25)	51 (78)	241	25 (59) [1]	182	20 (37)	18 (33)	62 (112)
Peach	193	18 (35) [1]	157	12 (19)	27 (42)	61 (96)	241	15 (35) [1]	206	15 (31)	22 (46)	63 (129)
Grapes	193	1 (2)	191	4 (7)	17 (33)	79 (151)	241	1 (2)	239	6 (15)	14 (34)	80 (190)
Cauliflower	192	4 (7)	185	23 (43)	20 (36)	57 (106)	240	5 (12)	228	26 (59)	27 (61)	47 (108) [2]
Zucchini	192	14 (27) [1]	165	58 (95)	22 (36)	21 (34) [2]	240	25 (61) [1]	179	54 (97)	27 (48)	19 (34) [2]
Carrot	191	1 (2)	189	16 (30)	24 (45)	60 (114)	241	0 (1)	239	16 (38)	27 (65)	57 (136)
Cucumber	191	1 (1)	190	6 (11)	11 (20)	84 (159)	240	0 (1)	239	6 (14)	9 (22)	85 (203)
Bell pepper	192	4 (7)	184	21 (38)	15 (28)	64 (118)	239	2 (5)	234	20 (47)	12 (27)	68 (160)
Broccoli	192	2 (3)	189	21 (39)	25 (48)	54 (102)	241	3 (6)	235	20 (48)	22 (52)	57 (135)
Green beans	192	6 (11)	181	25 (45)	31 (56)	44 (80) [2]	241	6 (15)	226	29 (65)	26 (59)	45 (102) [2]
Eggplant	192	43 (82) [1]	110	72 (79)	11 (12)	17 (19) [2]	241	52 (124) [1]	116	64 (74)	26 (30)	10 (12) [2]
Kale	192	20 (38) [1]	154	34 (52)	25 (39)	41 (63) [2]	241	24 (58) [1]	183	34 (62)	26 (48)	40 (73) [2]
Brussels sprouts	193	9 (17)	176	52 (91)	14 (24)	35 (61) [2]	240	9 (21)	219	53 (116)	18 (39)	29 (64) [2]
Leek	191	16 (30) [1]	161	37 (59)	30 (48)	34 (54) [2]	239	18 (43) [1]	196	35 (68)	34 (67)	31 (61) [2]
Tomato	193	2 (3)	190	33 (62)	12 (22)	56 (106)	240	4 (9)	231	27 (63)	10 (23)	63 (145)
Lettuce	193	1 (2)	191	16 (30)	26 (50)	58 (111)	240	3 (6)	234	16 (38)	19 (44)	65 (152)
Spinach	193	4 (7)	186	25 (46)	20 (38)	55 (102)	238	7 (17)	221	29 (63)	19 (41)	53 (117)
Onion	193	7 (13)	180	39 (70)	32 (57)	29 (53) [2]	238	8 (18)	220	35 (77)	31 (67)	35 (76) [2]
Peas	193	11 (22) [1]	171	26 (45)	27 (46)	47 (80) [2]	239	14 (33) [1]	206	28 (57)	23 (47)	50 (102) [2]

Note. Preference values were handled as missing values for participants who indicated to be unfamiliar with the specific FV item and for participants who selected >1 answer option.
Abbreviations: HPSF, Healthy Primary School of the Future.
[1] ≥10% participants indicated to be unfamiliar with the FV item. [2] ≤50% participants indicated to like the FV item.
