# Supplementary material for: The Effects of the Healthy Primary School of the Future on Children’s Fruit and Vegetable Preferences, Familiarity and Intake
Source: Nutrients. 2021 Sep 17;13(9):3241. doi: 10.3390/nu13093241 (PMC8469863; doi:10.3390/nu13093241)
Supplement: Supplementary file 1 [file nutrients-13-03241-s001.zip › Table S3. Observed mean fruit and vegetable intake at the various time points (T0-T4)..pdf]

Table S3. Observed mean fruit and vegetable intake at the various time points (T0-T4).

	**T0**					**T1**					**T2**				
**Outcome**	**Full HPSF**		**Partial HPSF**			**Full HPSF**		**Partial HPSF**			**Full HPSF**		**Partial HPSF**		
	n	% (n)	n	% (n)	***p*** †	n	% (n)	n	% (n)	***p*** †	n	% (n)	n	% (n)	***p*** †
Fruit intake	190		213		0·298	256		277		0·586	246		282		0·315
(almost) Never		6 (11)		5 (10)			4 (9)		4 (12)			5 (11)		6 (17)	
Sometimes (1-3 days/week)		16 (31)		24 (51)			22 (57)		21 (57)			24 (59)		23 (66)	
Often (4-6 days/week)		33 (62)		30 (63)			36 (93)		32 (89)			39 (96)		32 (91)	
Every day		45 (86)		42 (89)			38 (97)		43 (119)			33 (80)		38 (108)	
Vegetable intake	184		196		0·170	230		270		0·656	230		268		0·006 *
(almost) Never		4 (8)		4 (7)			7 (15)		4 (11)			5 (11)		8 (20)	
Sometimes (1-3 days/week)		12 (22)		21 (40)			14 (31)		13 (35)			10 (23)		18 (48)	
Often (4-6 days/week)		39 (72)		36 (71)			40 (93)		43 (115)			40 (91)		42 (112)	
Every day		45 (82)		40 (78)			40 (91)		40 (109)			46 (105)		33 (87)	

	**T3**					**T4**				
**Outcome**	**Full HPSF**		**Partial HPSF**			**Full HPSF**		**Partial HPSF**		
	n	% (n)	n	% (n)	***p*** †	n	% (n)	n	% (n)	***p*** †
Fruit intake	241		281		0·259	184		234		0·268
(almost) Never		5 (12)		3 (7)			7 (12)		3 (8)	
Sometimes (1-3 days/week)		20 (47)		25 (69)			20 (37)		18 (41)	
Often (4-6 days/week)		32 (77)		29 (82)			36 (67)		35 (81)	
Every day		44 (105)		44 (123)			37 (68)		44 (104)	
Vegetable intake	220		277		0·674	173		223		0·364
(almost) Never		4 (9)		7 (18)			4 (7)		3 (6)	
Sometimes (1-3 days/week)		14 (31)		13 (35)			11 (19)		13 (29)	
Often (4-6 days/week)		42 (92)		42 (116)			42 (73)		49 (109)	
Every day		40 (88)		39 (108)			43 (74)		35 (79)	

†Analyzed by X2 test.

Abbreviations: HPSF, Healthy Primary School of the Future.

* Significant (≤0·05) difference between full and partial HPSF without correction for baseline.
