# Supplementary material for: The Effects of the Healthy Primary School of the Future on Children’s Fruit and Vegetable Preferences, Familiarity and Intake
Source: Nutrients. 2021 Sep 17;13(9):3241. doi: 10.3390/nu13093241 (PMC8469863; doi:10.3390/nu13093241)
Supplement: Supplementary file 1 [file nutrients-13-03241-s001.zip › Table S4. Estimated intervention effects on fruit and vegetable familiarity as examined by a post hoc analysis using new summary scores..pdf]

Table S4. Estimated intervention effects on fruit and vegetable familiarity as examined by a post hoc analysis using new summary scores.

		**Full HPSF vs. partial HPSF**		
		**B (95% CI)**	***p***	**ES**
**Number of unfamiliar fruit items**	**T0-T1**	-0·183 (-0·418; 0·051)	0·126	-0·15
	**T0-T2**	0·040 (-0·226; 0·307)	0·766	0·03
	**T0-T3**	-0·058 (-0·344; 0·229)	0·692	-0·05
	**T0-T4**	0·349 (0·035; 0·663)	0·030*	0·29
**Number of unfamiliar vegetable items**	**T0-T1**	-0·554 (-0·957; -0·151)	0·007*	-0·29
	**T0-T2**	-0·512 (-0·970; -0·054)	0·028*	-0·27
	**T0-T3**	-0·733 (-1·224; -0·243)	0·003*	-0·38
	**T0-T4**	-0·459 (-0·992; 0·075)	0·092	-0·24

Note. In the present post hoc analysis, any fruit or vegetable item which was unfamiliar for <5% of the population at T0 was excluded from the new summary scores, resulting in the exclusion of apple, banana, tangerine, pear, strawberry, and grapes from the fruit summary score, and carrot, cucumber, broccoli, tomato, and lettuce from the vegetable summary score.
Note. Analyzed by linear mixed model analyses. All analyses were adjusted for sex, study year at T0, SES and ethnicity.
Abbreviations: HPSF, Healthy Primary School of the Future; CI, confidence interval; ES, effect size.
* Significant (≤0·05) difference between full and partial HPSF without correction for baseline.
