# Supplementary material for: The Effects of the Healthy Primary School of the Future on Children’s Fruit and Vegetable Preferences, Familiarity and Intake
Source: Nutrients. 2021 Sep 17;13(9):3241. doi: 10.3390/nu13093241 (PMC8469863; doi:10.3390/nu13093241)
Supplement: Supplementary file 1 [file nutrients-13-03241-s001.zip › Table S5. Estimated intervention effects on vegetable familiarity and preferences as examined by a post hoc analysis..pdf]

Table S5. Estimated intervention effects on vegetable familiarity and preferences as examined by a post hoc analysis.

			Full HPSF vs. partial HPSF		
			**B (95% CI)**	***p***	**ES**
**Familiarity**	**Number of unfamiliar vegetable items**	**T0-T1**	-0·497 (-0·841; -0·153)	0·005*	-0·31
		**T0-T2**	-0·321 (-0·716; -0·073)	0·110	-0·20
		**T0-T3**	-0·530 (-0·953; -0·108)	0·014*	-0·33
		**T0-T4**	-0·249 (-0·707; 0·210)	0·288	-0·16
**Preferences**	**Number of disliked vegetable items**	**T0-T1**	-1·684 (-2·558; -0·810)	0·000*	-0·36
		**T0-T2**	-1·043 (-2·068; -0·017)	0·046*	-0·23
		**T0-T3**	-0·059 (-1·177; 1·060)	0·918	-0·01
		**T0-T4**	-0·687 (-1·915; 0·541)	0·273	-0·15

Note. In the present post hoc analysis, vegetable items were excluded if children were not exposed to them between T0-T4 as part of the HPSF intervention. This resulted in the exclusion of Brussels sprouts, broccoli, cauliflower, green beans, and kale from the vegetable familiarity and preferences summary score.
Note. Analyzed by linear mixed model analyses. All analyses were adjusted for sex, study year at T0, SES and ethnicity.
Abbreviations: HPSF, Healthy Primary School of the Future; CI, confidence interval; ES, effect size.
* Significant (≤0·05) difference between full and partial HPSF.
